# 血清TPS、CEA、Pro-GRP和CYFRA21-1水平在肺癌患者中的临床意义

**DOI:** 10.3779/j.issn.1009-3419.2010.05.22

**Published:** 2010-05-20

**Authors:** 敬慧 王, 广利 时, 树才 张, 群慧 王, 新杰 杨, 曦 李, 海永 王, 卉 张, 长兴 宋

**Affiliations:** 1 101149 北京，北京胸科医院肿瘤内科 Department of Medical Oncology, Beijing Chest Hospital, Beijing 101149, China; 2 101149 北京，北京胸科医院临床免疫室 Department of Clinical Immunology Laboratory, Beijing Chest Hospital, Beijing 101149, China

**Keywords:** 肺肿瘤, 组织多肽特异性抗原, 诊断, 预后, Lung neoplasms, Tissue polypeptide specifc antigen, Diagnosis, Prognosis

## Abstract

**背景与目的:**

血清肿瘤标志物在肺癌的诊断、疗效、预后判断中起着重要作用。本研究探讨血清组织多肽特异性抗原（tissue polypeptide specifc antigen, TPS）与癌胚抗原（carcinoembryonic antigen, CEA）、胃泌素释放肽前体（precursor of gastrin-releasing peptide, Pro-GRP）和细胞角蛋白19片段（cytokeratin-19-fragments, CYFR21-1）的水平及其在肺癌患者中的临床意义。

**方法:**

应用ELISA检测82例肺癌患者化疗前及部分患者化疗后4种标志物水平。

**结果:**

肺癌患者TPS、CEA、Pro-GRP阳性率及水平显著高于肺部良性疾病组和健康对照组。广泛期小细胞肺癌患者TPS阳性率显著高于局限期患者。患者化疗后TPS、CEA、Pro-GRP阳性率及水平均显著下降。非小细胞肺癌患者TPS水平是预后的独立因素。

**结论:**

TPS在肺癌患者的辅助诊断、疗效观察有较好的临床意义，对非小细胞肺癌的预后判断方面可能有一定价值。

## Abstract (en)

**Background and objective:**

Serum tumor markers play important roles in diagnosis, response and prognosis monitoring for lung cancer. The clinical signifcance of serum level of tissue polypeptide specifc antigen (TPS) was investigated in diagnosis, response monitoring and prognosis in patients with lung cancer, compared with carcinoembryonic antigen (CEA), precursor of gastrin-releasing peptide (Pro-GRP) and cytokeratin-19-fragments (CYFRA21-1).

**Methods:**

Blood samples of eighty-two patients with lung cancer before treatment and some after chemotherapy were measured by ELISA for four tumor markers.

**Results:**

Compared with lung benign diseases group and health control group, the positive rates and levels of TPS, CEA and Pro-GRP in patients with lung cancer were higher, with statistically signifcant difference. TPS in extensive-small cell lung cancer was signifcant higher than that in limited-small cell lung cancer. The positive rates and levels of TPS, CEA and Pro-GRP in patients afer treatment had signifcant decreases compared with before treatment. TPS was an independent prognostic factor of non-small cell lung cancer.

**Conclusion:**

TPS is valuable to diagnosis, response monitoring for patients with lung cancer, moreover, it maybe a useful factor of prognosis of non-small cell lung cancer.

肺癌是当前癌症相关死亡的首要原因，包括非小细胞肺癌（non-small cell lung cancer, NSCLC）和小细胞肺癌（small cell lung cancer, SCLC），肺癌患者早期诊断率低，确诊时大多数为晚期，远期生存差。恰当的血清肿瘤标准物在肿瘤的筛查、诊断、疗效监测、预后判断等方面有重要的临床应用价值。组织多肽特异性抗原（tissue polypeptide specific antigen, TPS）是细胞角蛋白18片段上的M3抗原决定簇，能够反映肿瘤细胞分裂增殖活性，常被用于前列腺癌、乳腺癌、卵巢癌等的诊断及预后，已被国外学者广泛应用于肺癌患者中。本文前瞻性研究TPS在肺癌患者中的临床意义，同时与常用的标志物癌胚抗原（carcinoembryonic antigen, CEA）、胃泌素释放肽前体（precursor of gastrin-releasing peptide, Pro-GRP）和细胞角蛋白19片段（cytokeratin-19-fragments, CYFRA21-1）做比较，评价它们在肺癌患者诊断、疗效监测及预后判断方面的作用。

## 1 材料与方法

### 1.1 研究对象

2007年4月-2008年6月期间在我院住院的肺癌患者82例，全部经细胞学或组织学确诊，既往未行放化疗。男性55例，女性27例，年龄29岁-80岁，中位年龄55岁，平均55.95岁。NSCLC患者61例，其中腺癌41例，鳞癌20例；Ⅲa期3例，Ⅲb期20例，Ⅳ期38例；SCLC患者21例，局限期9例，广泛期12例；全部患者经体格检查、胸片、腹部彩超、头胸CT、骨扫描等确定临床分期，小细胞肺癌患者同时行骨髓穿刺。NSCLC分期参照2002年AJCC/UICC肺癌分期标准，SCLC分期参照美国退伍军人医院的肺癌分期标准。肺部良性疾病组24例，为本院确诊的肺炎或肺结核患者，健康对照组为本院体检过的30例健康职工。26例NSCLC（鳞癌7例，腺癌19例）和18例SCLC在化疗2周期后再次检测4种标志物水平（表 1）。

**1:** 患者一般资料 Patient characteristics

Characteristics	All (*n*=82)	After treatment (*n*=44)	*P*
Gender			0.899
Male	55	30	
Female	27	14	
Age (yrs)			0.601
Median	55	56	
Range	29-80	29-74	
Non-small cell lung cancer			0.588
Adenocarcinoma	41	19	
Squamous	20	7	
Performance status (ECOG)			0.080
0	7	5	
1	37	19	
2	9	2	
3	6	0	
4	2	0	
Stage			0.496
Ⅲa	3	1	
Ⅲb	20	12	
Ⅳ	38	13	
Small cell lung cancer	21	18	
Stage			0.921
Limited disease	9	8	
Extensive disease	12	10	
Performance status (ECOG)			0.600
0	2	2	
1	11	11	
2	6	5	
3	2	0	

### 1.2 标本采集及检测

全部患者在化疗前抽取空腹静脉血4 mL，部分患者在化疗2周期后再次抽血。标本分离出血清，-20 ℃保存，集中进行检测。采用ELISA方法检测TPS、CEA、Pro-GRP和CYFRA21-1，试剂盒分别为瑞典IDL Biotech AB公司、郑州安图绿科生物工程有限公司、日本三株社会生物公司、美国ADL公司生产。检测严格参照各试剂盒的说明书操作。TPS、CEA、Pro-GRP、CYFRA21-1的临界值分别为80 U/L、5 ng/mL、50 pg/mL和4 ng/mL，检测值超过临界值为阳性。

### 1.3 化疗方案及疗效评价

全部患者的血常规、肝肾功能及体力状况评分等指标均符合化疗要求，化疗2周期后进行疗效评价。参照WHO实体瘤疗效评价标准：完全缓解（complete response, CR）、部分缓解（partial response, PR）、疾病稳定（stable disease, SD）和疾病进展（progressive disease, PD），CR及PR患者需4周确认。对全组患者进行随访，记录生存期，随访日期截止至2009年6月30日。

### 1.4 统计学方法

数据进行正态检验，非正态分布数据采用非参数检验。阳性率的比较采用*χ*^2^检验或*Fisher’s*检验。不同组间标志物水平差异采用*Mann-Whitney U*检验；治疗前后标志物水平差异采用*Wilcoxon*秩和检验；用*Spearman’s*相关检验评价不同标志物间的相关性；生存分析采用*Kaplan-Meier*法，两组间生存差异分析采用*Logrank*检验；*Cox*比例风险回归模型分析多种因素对预后的影响。数据分析及绘图由SPSS 10.0统计软件完成，*P* < 0.05为差异有统计学意义。

## 2 结果

### 2.1 化疗前标志物阳性率及水平

#### 2.1.1 三组间标志物比较

肺癌组TPS、CEA、Pro-GRP阳性率及水平均显著高于肺部良性疾病组和健康对照组，三组间CYFRA21-1阳性率及水平无统计学差异（表 2）。

**2:** 肺癌组、肺部良性疾病组、健康对照组TPS、CEA、Pro-GRP和CYFRA21-1治疗前的比较 Pre-treatment comparison of TPS, CEA, Pro-GRP and CYFRA21-1 in patients with lung cancer, pulmonary benign diseases and health control

Group	*n*	TPS			CEA			Pro-GRP			CYFRA21-1	
		*n* (%)	Concentration (U/L)		*n* (%)	Concentration (ng/L)		*n* (%)	Concentration (pg/mL)		*n* (%)	Concentration (ng/mL)
Lung caner	82	45 (54.9)^*#^	149.25±130.97^*#^		25 (30.5)^**#^	13.54±22.12^**##^		16 (19.5)^**##^	119.63±267.70^**##^		5 (6.1)^***###^	1.01±1.21^***###^
Pulmonary benign diseases	24	1 (3.3)	52.31±21.65		0 (0)	1.33±1.19		0 (0)	11.12±10.89		0	0.88±0.71
Health control	30	1 (3.3)	47.44±19.21		0 (0)	1.55±1.17		0 (0)	10.44±9.83		0	0.67±0.56
Compared with pulmonary benign diseases group: ^*^P<0.001, ^**^P<0.05, ^***^P>0.05; Compared with health control group: ^#^*P*<0.001, ^##^*P*<0.05, ^###^*P*>0.05.												

#### 2.1.2 不同病理类型间标志物比较

TPS阳性率在三种病理类型之间无统计学差异，鳞癌CYFRA21-1阳性率显著高于腺癌和SCLC，SCLC Pro-GRP阳性率显著高于腺癌和鳞癌，腺癌CEA阳性率显著高于鳞癌，其它两两比较无统计学差异。鳞癌和SCLC的TPS水平均显著高于腺癌，腺癌CEA水平分别显著高于鳞癌和SCLC，SCLC的Pro-GRP水平分别显著高于腺癌和鳞癌，三种病理类型间CYFRA21-1水平无统计学差异（表 3）。

**3:** 不同病理类型患者TPS、CEA、Pro-GRP和CYFRA21-1的比较 Pre-treatment comparison of TPS, CEA, Pro-GRP and CYFRA21-1 in patients with different histologic types

Group	*n*	TPS			CEA			Pro-GRP			CYFRA21-1	
		*n* (%)	Concentration (U/L)		*n* (%)	Concentration (ng/L)		*n* (%)	Concentration (pg/mL)		*n* (%)	Concentration (ng/mL)
Squamous	20	14 (70.0)^*^	177.75±137.94^*^		3 (15.0)^*^	5.06±10.60^*^		1 (5.0)^**^	47.90±118.85^**^		4 (20.0%)^**^	1.55±2.22^*^
Small cell lung cancer	21	12 (57.1)^#^	181.67±119.71^##^		4 (19.0)^#^	9.29±20.21^#^		14 (66.7)^##^	360.71±416.65^##^		0 (0)^#^	0.78±0.89^#^
Adenocarcinoma	41	19 (46.3)^△^	122.91±131.39^△△^		18 (43.9)^△△^	18.62±24.82^△△^		1 (2.4)^△^	20.65±25.00^△^		1 (2.4%)^△△^	1.01±1.16^△^
Compared squamous with small cell lung cancer: ^*^P>0.05, ^**^P < 0.05; compared small cell lung cancer with adenocarcinoma: ^#^*P*>0.05, ^##^*P* < 0.05; compared adenocarcinoma with squamous: ^△^*P*>0.05, ^△△^*P* < 0.05.												

#### 2.1.3 不同分期间标志物比较

SCLC Ⅲb期和Ⅳ期患者TPS、CEA、CYFRA21-1阳性率均无统计学差异。广泛期SCLC患者TPS阳性率显著高于局限期患者（*P*=0.040），CEA、Pro-GRP在两种分期间无统计学差异。

### 2.2 标志物与疗效的关系

26例NSCLC患者化疗2周期后，CR 0例，PR 9例，SD 14例，PD 3例，与化疗前水平比较，PR+SD患者TPS、CEA水平均显著下降。5例治疗后TPS增高患者中，其中3例数值较化疗前明显下降，另外2例为治疗前正常疗效为PD的患者，这2例患者CEA未见增高。NSCLC患者化疗前后CYFRA21-1无统计学差异。18例SCLC患者中，CR 1例，PR 14例，SD 3例，患者的TPS、Pro-GRP水平均有显著下降。少数患者化疗后TPS、CEA、Pro-GRP水平仍异常，但较治疗前有明显下降。化疗后NSCLC患者TPS阳性率及SCLC患者TPS、Pro-GRP阳性率均有明显下降（表 4，表 5）。

**4:** 肺癌患者TPS、CEA、Pro-GRP和CYFRA21-1与疗效的关系 Relationships between response and TPS, CEA, Pro-GRP and CYFRA21-1 in patients with lung cancer without PD

	Pretreatment	Without PD	*P*
NSCLC			
TPS			0.001
Normal	10	20	
Elevated	16	3	
CEA			0.390
Normal	15	16	
Elevated	11	7	
CYFRA21-1			0.237
Normal	23	23	
Elevated	3	0	
SCLC			
TPS			0.002
Normal	7	16	
Elevated	11	2	
Pro-GRP			0.001
Normal	6	16	
Elevated	12	2	

**5:** 肺癌患者治疗前后TPS、CEA、Pro-GRP和CYFRA21-1水平的比较 TPS, CEA, Pro-GRP and CYFRA21-1 comparison before and after treatment in patients with lung cancer without PD

Group	TPS concentration (U/L)	CEA concentration (ng/L)	Pro-GRP concentration (pg/mL)	CYFRA21-1 concentration (ng/mL)
NSCLC				
Before treatment	111.02±100.28	16.04±25.25	14.91±13.82	1.34±1.01
After treatment	47.97±32.19^#^	6.97±11.36^#^	23.17±43.99^##^	0.90±1.16^##^
SCLC				
Before treatment	192.54±124.02	10.42±21.67	322.58±401.76	0.76±0.78
After treatment	38.59±30.78^#^	2.58±5.26^##^	33.52±73.42^#^	0.68±0.72^##^
Comparison of concentration: ^#^*P* < 0.05，^##^*P* > 0.05.				

### 2.3 标志物之间的相关关系

NSCLC患者中，TPS与CEA无相关性（r_s_=-0.039, *P*=0.779），TPS与CYFRA21-1无相关性（r_s_=0.183, *P*=0.312），CEA与CYFRA21-1无相关性（r_s_=0.143, *P*=0.298）；SCLC患者中，TPS、CEA无相关性（r_s_=0.221, *P*=0.336），TPS与Pro-GRP间无相关性（r_s_=-0.374, *P*=0.095），Pro-GRP与CEA无相关性（r_s_=-0.157, *P*=0.496）。

### 2.4 受试者工作特征曲线（receiveroperating characteristic, ROC）

ROC曲线分析显示，NSCLC曲线下面积分别为：TPS为0.845±0.042，CEA为0.751±0.051，Pro-GRP为0.618±0.060，CYFRA21-1为0.364±0.061，TPS曲线下面积最大，其次为CEA（图 1）。

**1:**
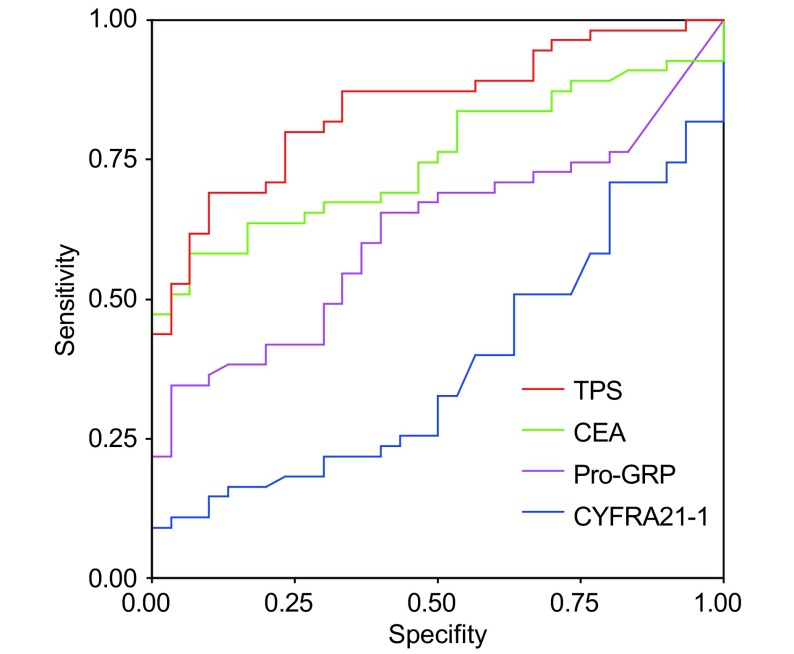
非小细胞肺癌的ROC曲线 ROC curves analysis in NSCLC

SCLC患者曲线下面积分别为：TPS为0.936±0.037，Pro-GRP为0.814±0.075，CEA为0.450±0.090，CYFRA21-1为0.292±0.075，TPS曲线下面积最大，其次为Pro-GRP（图 2）。

**2:**
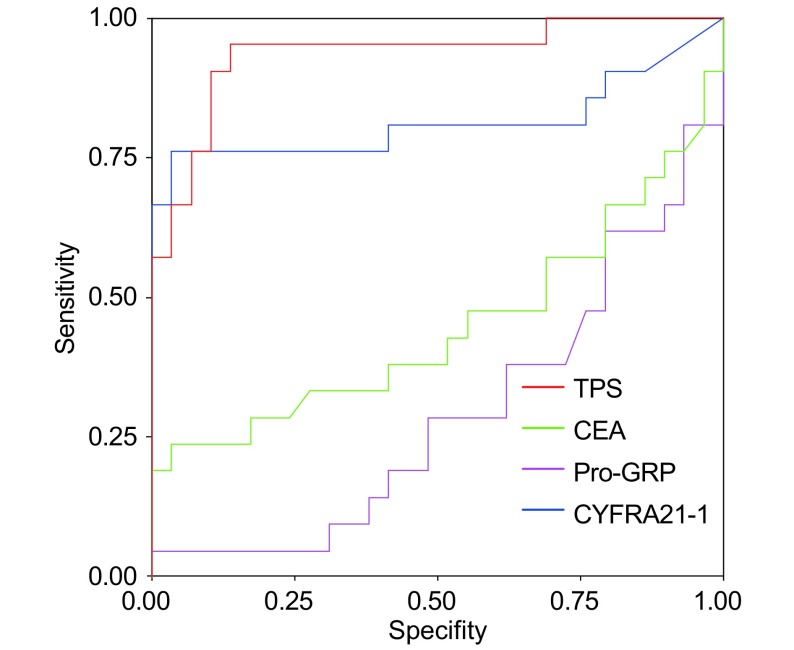
小细胞肺癌的ROC曲线 ROC curves analysis in SCLC

### 2.5 预后因素分析

NSCLC患者中位生存期为10个月（范围为2个月-18个月），SCLC患者中位生存期为13个月（范围为6个月-18个月）。将PS评分、治疗前TPS、CEA、CYFRA21-1水平作为分析变量对NSCLC患者生存进行*Cox*多因素回归分析，PS、TPS是NSCLC影响患者预后的独立因素。将分期、TPS、Pro-GRP作为分析变量对SCLC患者生存进行*Cox*多因素回归分析，分期（*P*=0.030）是影响SCLC患者预后的独立因素（图 3，表 6）。

**3:**
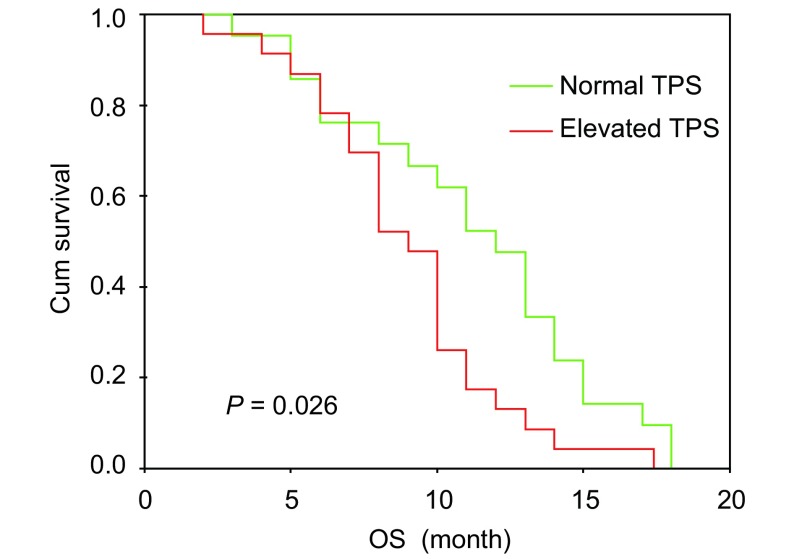
治疗前TPS水平与NSCLC患者生存期 NSCLC survival depending on the pretreatment TPS level

**6:** 肺癌患者生存多因素分析 *Cox* analysis of survival in patients

Variables and levels	Median survival time (month)	*P*
Non-small cell lung cancer		
PS		0.021
0-1	14	
2	6	
TPS		0.026
Normal	13	
Elevated	9	
CEA		0.364
Normal	11	
Elevated	10	
CYFRA21-1		0.526
Normal	10	
Elevated	11	
Small cell lung cancer		
Stage		0.030
Limited disease	14	
Extensive disease	11	
TPS		0.849
Normal	12	
Elevated	11	
Pro-GRP		0.600
Normal	12	
Elevated	12	

## 3 讨论

本文通过对82例肺癌患者血清4种标志物的分析，发现肺癌患者血清TPS、CEA、Pro-GRP阳性率显著高于肺部良性疾病患者及健康对照者，有统计学差异，阳性率依次为54.9%、30.5%、19.5%，TPS最高，三组间CYFRA21-1无差异，可能与本组患者鳞癌例数相对不大有关。TPS在鳞癌、SCLC、腺癌患者的阳性率分别为70.0%、57.1%、46.3%，鳞癌患者阳性率最高，三者无差异，显示TPS在各种类型肺癌中的阳性率均较高。CYFRA21-1、Pro-GRP和CEA等是临床上广泛应用的肺癌标志物，分别对鳞癌、SCLC及腺癌有较高的诊断价值。本研究鳞癌患者TPS阳性率明显高于CYFRA21-1（71.4% *vs* 20.0%），显示TPS在鳞癌的诊断价值明显优于CYFRA21-1。本组SCLC患者TPS阳性率虽然低于Pro-GRP（57.1% *vs* 66.7%），但阳性率仍较高。本组腺癌患者TPS阳性率略高于CEA（46.3% *vs* 42.9%），二者相近。本研究结果表明TPS在鳞癌、SCLC及腺癌患者中均具有良好的诊断价值，与国内外研究一致^[1, 2]^。

标志物水平常与肺癌患者的分期密切相关，分期越晚的患者标志物水平越高。本研究发现，NSCLC不同分期间患者TPS、CEA、CYFRA21-1无差异，这与本组NSCLC患者绝大多数为Ⅲb期及Ⅳ期有关。广泛期SCLC患者TPS阳性率显著高于局限期患者，有统计学差异，证实TPS能够反映SCLC患者的疾病严重程度，在判断病情方面有价值，与李学祥等^[3]^的研究一致。而Pro-GRP水平与分期无关，其在局限期患者中也具有较好的诊断意义，适合在肿瘤筛查中使用。

肿瘤标志物最重要的作用之一是监测疗效。本研究对26例NSCLC和18例SCLC患者化疗2周期后再次采集静脉血，对4种标志物进行检测，结果发现，NSCLC患者TPS、CEA水平显著下降，5例治疗后TPS异常的患者中，3例治疗前异常，但数值较治疗前有明显下降，另外2例为疗效PD的患者，他们治疗前的TPS正常，这说明TPS能够很敏感地反映机体肿瘤负荷的变化，而这2例PD患者的CEA未见增高。18例SCLC患者化疗2周期后无PD患者，TPS、Pro-GRP均有显著下降，化疗后仍有2例TPS、Pro-GRP异常，但数值较治疗前也有明显下降。本研究显示病情好转或稳定的肺癌患者TPS、CEA、Pro-GRP治疗后水平显著下降，表明肿瘤细胞产生明显减少，与病情好转一致。虽然CEA治疗前后水平有显著下降，但阳性率无差异，PD患者数值也无升高，说明TPS较CEA更能体现NSCLC患者病情变化，TPS及Pro-GRP均能很好地反映SCLC患者的治疗效果，对临床判断疗效有实际意义，上述结果与文献^[1, 3, 4]^报告一致。

本研究对标志物相关性进行了分析，结果显示，NSCLC患者TPS、CEA和CYFRA21-1三者间两两均无相关性，SCLC患者TPS、CEA、Pro-GRP之间也无相关性。TPS与CYFRA21-1来源相似，本文未观察到二者有相关性，其它几种标志物也无相关性，说明这几种肿瘤标志物各自独立，这为联合检测提供理论基础。

ROC曲线是一种全面、准确评价诊断试剂的非常有效的方法，通过ROC曲线下面积，可判断肿瘤标志物的诊断效率。本研究ROC曲线显示，NSCLC和SCLC患者的TPS曲线下面积均为最大，分别高于CEA、Pro-GRP，显示TPS对NSCLC和SCLC患者均具有很好的诊断价值。

许多研究^[5-7]^显示TPS及CYFRA21-1是NSCLC独立的预后因素，Pro-GRP能够反映SCLC患者的预后。本研究Cox多因素分析显示，PS评分、TPS是NSCLC的独立预后因素，而CEA、CYFRA21-1与NSCLC患者的预后无关，表明TPS在预测NSCLC生存方面优于其它标志物。分期是SCLC患者预后的独立因素，而TPS、Pro-GRP与患者预后无关，本组SCLC患者样本量偏小，值得扩大样本进一步研究。

综上所述，本文研究结果显示TPS对肺癌有很好的诊断价值，能够反映疗效，并对NSCLC的预后有判断价值，值得在临床上推广使用。
